# The Future of Spinal Anæsthesia

**Published:** 1910-01-29

**Authors:** 


### The Future of Spinal Anæsthesia.

The recent visit of Professor Jonnesco, of Bucharest, to this country, and his demonstrations of the complete anæsthesia to be obtained by injecting into the spinal canal solutions of stovaine combined with strychnine, have once more drawn attention to the claims of spinal anæsthesia in general and the Jonnesco method in particular. And, indeed, it is high time that the profession, as a whole, became conscious of the merits of these new methods of inducing general analgesia, and that some pains were taken to arrive at an accurate estimate of the relative value of the Jonnesco compared with other methods. The marked interest that the demonstrations at the Seamen's Hospital aroused shows how little the profession in this country knew about methods which have been in use in Continental operating-rooms for the last two years, and the fact that many surgeons, both in private and in hospital practice, refuse to try the new methods, while others, again, look askance at them and only try them in extreme and very often unsuitable cases, is an added proof that spinal anæsthesia has not yet come into its own in this country.

The reasons for this prejudice in favour of general anæsthesia by inhalation methods are too varied and diverse to be enumerated within the compass of a brief article. One of the main causes of the prejudice against spinal anæsthesia is ignorance of the technique and want of knowledge of the particular conditions in which it is suitable. It is a grave mistake to suppose that spinal anæsthesia is indicated in cases where an expert anæsthetist would hesitate to give a general anæsthetic. To give it in such cases is to court disaster and to bring discredit upon a method which is really sound and efficacious in properly selected cases. While it has been proved that spinal anæsthesia is quite successful in operations on the upper extremity—the thorax, head, face, and neck—it is nevertheless doubtful whether the Jonnesco method in such cases will ever be popular with English surgeons or, what is much more to the point, with English patients. It has been repeatedly pointed out that the mental attitude of the patient is a very considerable factor in cases of injection analgesia. Most practitioners can recall one or more cases in their own practice in which grave symptoms have followed a simple operation performed under cocaine, and as the literature of spinal anæsthesia increases instances of dangers and disasters produced by the patient's nervousness or excitability are frequently reported. In general anæsthesia the patient is unconscious; he can offer no help to the operator, but at the same time he cannot embarrass the operation by struggling, nervous starts, or even by fainting. While every effort is, of course, taken to prevent the patient seeing the actual steps of the operation, it is almost impossible to arrange matters so that he cannot hear the sounds of the instruments and the talk of the assistants.

Another obvious objection to the use of spinal anæsthesia is the uncertainty that exists with regard to its after effects. The statistics of spinal methods are by no means easy to obtain, scattered as they are in the literature, and they still need careful collation and editing. This largely accounts for the diversity of opinion with regard to the safety of spinal injections for inducing general analgesia. In England, where comparatively small doses of stovaine or novocaine are employed, the percentage mortality has been high—perhaps unduly so—but at all events high enough to make some surgeons chary of employing spinal analgesia under any conditions. With the use of the less toxic tropacocaine and the Jonnesco method, in which the addition of strychnine counteracts the fall of blood-pressure, the percentage mortality has been very much reduced, but, to judge from published statistics, it is still higher than with ether. Then, again, there are the after-effects to be considered, and while it is generally agreed that these are not serious if proper care is taken and suitable cases are selected, the practitioner will prefer to wait until he has fuller information about their possibilities than is available at present. In the circumstances, spinal anæsthesia is a subject which the expert anæsthetist might well investigate at length, and about which he might usefully publish all information that he is able to obtain.

